# How to interpret patient-reported outcomes? - Stratified adjusted minimal important changes for the EQ-5D-3L in hip and knee replacement patients

**DOI:** 10.1186/s41687-024-00812-x

**Published:** 2024-11-25

**Authors:** Irene Salvi, David Ehlig, Justus Vogel, Anja Bischof, Alexander Geissler

**Affiliations:** 1 Chair of Health Economics, Policy, and Management, School of Medicine, University of St. Gallen, St. Jakob-Strasse 21, St. Gallen, SG 9000 Switzerland; 2 Chair of Digital Health Interventions, School of Medicine, University of St. Gallen, St. Gallen, SG Switzerland

**Keywords:** Minimal important changes, Adjusted minimal important changes, Patient-reported outcomes, EQ-5D, Patient characteristics, Hip replacement, Knee replacement

## Abstract

**Background:**

As one of the main goals of hip and knee replacements is to improve patients’ health-related quality of life, a meaningful evaluation can be achieved by calculating minimal important changes (MICs) for improvements in patient-reported outcome measures (PROMs). This study aims at providing MICs adjusted for patient characteristics for EQ-5D-3L index score improvements after hip and knee replacements. It adds to existing literature by relying on a large national sample and precise clustering algorithms, and by employing a state-of-the-art methodology for the calculation of improved adjusted MICs.

**Methodology:**

A retrospective observational study was conducted using the publicly available National Health Service (NHS) PROMs dataset for primary hip and knee replacements. We used information on 252,331 hip replacements and 279,668 knee replacements from all NHS-funded providers in England between 2013 and 2020. Clusters of patients were created based on pre-operative EQ-VAS, depression status, and sex. Unstratified and stratified estimates for meaningful EQ-5D-3L improvements were obtained through anchor-based predictive MICs corrected for the proportion of improved patients and the reliability of transition ratings.

**Results:**

Stratifying patients showed that MICs varied across subgroups based on pre-operative EQ-VAS, depression status, and sex. MICs were larger for patients with worse pre-operative EQ-VAS scores, while patients with better pre-operative scores required smaller MICs to achieve a meaningful change. We show how after stratification the percentage of patients achieving their stratified MIC was better in line with the actual share of improved patients. Larger MICs were found for patients with depression and for female patients. MICs calculated for knee replacements were consistently lower than those for hip replacements.

**Conclusions:**

Our findings show the importance of adjusting MICs for patients’ characteristics and should be considered for quality-related choices and policy initiatives.

**Supplementary Information:**

The online version contains supplementary material available at 10.1186/s41687-024-00812-x.

## Background

Hip and knee replacements are two of the most frequently performed and effective orthopedic surgeries worldwide. They are usually performed once conservative treatment approaches have been tried and failed for end-stage osteoarthritis [1]. In 2019, roughly 101,384 hip replacements [2] and 108,506 knee replacements [3] were performed in the UK alone, and these numbers are set to increase over the next years [4].

As one of the main aims of hip and knee replacements is to improve patients’ health-related quality of life (HRQoL) [5, 6], it is important to measure HRQoL improvement after surgery with suitable instruments. Patients being the best judges of their own HRQoL, patient-reported outcome measures (PROMs) have been proposed as suitable instruments to understand whether surgeries such as hip or knee replacements were successful in terms of HRQoL [7, 8]. In particular, the generic instrument EQ-5D has been shown to be a valid instrument for the measurement of health-related quality of life after hip and knee replacement [9–11]. The use of this generic PROM has the advantage that it provides an assessment of the overall health status beyond joint-specific outcomes, encompassing a broader range of physical and mental health dimensions relevant to patients undergoing hip and knee replacement surgeries [11].

Over the years, patients have reported some degree of dissatisfaction with hip and knee replacement results, ranging from 20 to 7% for hip replacements [12, 13] and 29–13% for knee replacements [13, 14]. One possible approach to help offset negative surgery outcomes reported by patients and to set the right patients’ expectations is to investigate PROM results. To measure outcomes from the perspective of the patient and to encourage measurable improvements, in April 2009 the English National Health Service (NHS) began to collect PROMs on a yearly basis from all NHS-funded providers [15].

However, statistical significance of a PROM score change does not necessarily mean that such change is also clinically relevant [16–18]. Clinicians and health policy makers need to understand how to identify and interpret clinically meaningful results to correctly use them for clinical decision making and health policy development [19]. One way to do so is to calculate minimal important changes (MICs) of the score of interest. In this article, we adopt the definition provided by Terwee et al. [20] of MIC as “a threshold for a minimal within-person change over time above which patients perceive themselves importantly changed”.

However, MICs calculated for PROM scores of the average patient may lead to biased treatment evaluations [21]. By estimating MICs adjusted for patient characteristics, it is possible to show a more realistic estimation of the percentage of successful hip and knee replacements [22].

Previous studies have estimated MICs for the EQ-5D after hip or knee replacement [23, 24]. However, these studies are limited by sample size, lack of adjustment for patient characteristics, not fully suitable anchors, and/or imprecise clustering algorithms. Most studies also do not present a correction for the proportion of improved patients [25], and the reliability of transition ratings [26], which are necessary to avoid overestimation of MIC estimates in datasets with more than 50% of patients improved on the anchor.

Furthermore, to the best of our knowledge, no study has so far explored the impact of mental health and sex-specific effects in MICs.

With this study, we aim at estimating more precise MICs based on patients characteristics that have been highlighted in the literature as relevant for the correct interpretation of PROMs. Accordingly, we cluster patients based on pre-operative HRQoL status, mental health status, and sex [27–29]. We employ a larger sample size, a more reliable anchor and more precise clustering algorithms with respect to existing studies. Furthermore, we implement a correction for the proportion of improved patients and the reliability of transition ratings.

## Methods

### Data source

We conducted a retrospective observational study using the publicly available National Health Service (NHS) PROMs dataset on hip and knee replacements, with data from all NHS-funded providers in England between 2013 and 2020 and a response rate of more than 60% [30]. We excluded datasets from the years 2009 to 2012 due to the absence of distinction between primary replacements and revisions and the presence of fewer variables. See Table A1 for a detailed description of the data access procedure.

The STROBE Statement guidelines for reporting observational studies was followed [31].

### Variables

The dataset includes information on patient demographics, pre-operative health history, comorbidities, pre- and post-operative EQ-5D-3L, Oxford Hip Score (OHS) and Oxford Knee Score (OKS), and other post-operative information. The post-operative follow-up time was determined by the NHS to be 6 months [32]. 

The EQ-5D-3L is an internationally well-established generic PROM which takes into account five overall HRQoL dimensions, namely mobility, self-care, usual activities, pain/discomfort and anxiety/depression. Each dimension has three response levels and its scores are aggregated into a single index ranging from − 0.594 to 1 (1 being full health and 0 being the equivalent of death) for the UK population [33].

The OHS and OKS are joint-specific PROMs with values ranging from 0 (most severe symptoms) to 48 (least symptoms) designed to assess disability in patients undergoing hip replacement (knee replacement) [34, 35].

One question collected in the framework of the NHS PROMs dataset as part of the post-operative questionnaires is the so-called variable “Success”. This variable consists in the answers (namely, “Much better”, “A little better”, “About the same”, “A little worse”, and “Much worse”) to the question “Overall, how are your problems now, compared to before your operation?”. While we refer to it throughout the paper as the variable “Success” according to its original name in the NHS PROMs dataset, it should rather be intended as a variable representing whether the patients consider themselves to have (meaningfully) improved or not.

### Data cleaning and final data set

Between April 01, 2013, and March 31, 2020, 297,806 patients received hip replacements, of which we excluded 16,560 observations with the “Revision Flag”, as we are only interested in primary joint replacements. Of the remaining 281,246 primary hip replacements, 255,132 completed both the pre-operative and 6-month post-surgery follow-up EQ-5D-3L questionnaire. We excluded 617 cases for which the pre-operative EQ-5D-3L was already recorded as “full health” (corresponding to a score of 1.0), since no further score improvement was possible. 2,184 cases for which the anchor variable was not available were eliminated. Finally, we excluded 21,723 cases that were missing either the pre- or the post-operative EQ-VAS questionnaire, leaving 230,608 cases for the analysis. We performed data cleaning steps for the knee replacement sample analogue to the hip replacement sample, reaching a final data set of 254,508.

Before implementing the analyses, some variables in the dataset were transformed for a better representation in the model. For an illustration, see Figure A1 in the Appendix. See Table A2 in the Appendix for an overview of such changes.

### Empirical methods and approach

To characterize the sample, descriptive statistics were performed. It has been shown that stratification on the baseline score induces spurious baseline dependency of the MIC estimates [36]. Therefore, we first clustered the patients according to the pre-operative EQ-VAS as a measure of pre-operative HRQoL, as it presents an acceptable level of correlation with the EQ-5D-3L of 0.36 (0.34) for hip (knee) replacement patients. As a second stratification, we stratified by pre-operative Depression status, measured as a positive answer to the question “Have you been told by a doctor that you have depression?”, and by Depression status and pre-operative EQ-VAS. To test differences in MICs between male and female patients, a split according to the variable “Gender” (denoting the sex of the patient according to the NHS PROMs data dictionary [37]) and “Gender” and pre-operative EQ-VAS was implemented. The k-means algorithm was employed for univariate clustering for continuous variables, while a dual split was applied for binary variables.

Subsequently, we estimated the MICs for the unstratified and stratified samples. MICs have a long history dating back to their first mention in 1987 by Guyatt et al. [38]. In 1989, Jaeschke et al. [39] further developed the concept and defined the MCID as “the smallest difference in score in the domain of interest which patients perceive as beneficial and which would mandate […] a change in the patient’s management”. In this article, we adopt the definition by Terwee et al. [20] of “a threshold for a minimal within-person change over time above which patients perceive themselves importantly changed”. We focus here on the MICs that need to be achieved in order for the hip or knee replacement patients to be meaningfully improved.

In this study we calculated the anchor-based predictive MIC, by implementing the adjustment for the proportion improved and the transition rating reliability as proposed by Terluin et al. [26]. The advantage of the anchor-based approach is that change in the outcome measure score is linked to a meaningful external anchor that accounts for the patient’s perspective [40]. While the receiver operating characteristic (ROC) and predictive modeling methods are viable when the proportion improved is close to 0.5, both suffer from biases when this proportion deviates from 0.5. In such cases, the adjusted MIC method is recommended for its ability to correct these biases, providing a more accurate estimation of the genuine MIC [25]. Furthermore, the (un)reliability of transition ratings brings additional bias to the MIC estimation, particularly when the proportion of patients improved deviates from 0.5 [26]. To account for the biasing effects of the proportion of patients reporting improvement and the reliability of transition ratings, we employed the updated formula for the adjustment of the MIC. The formula refines the predictive MIC by subtracting a term that accounts for the bias introduced by the reliability of transition ratings, the standard deviation of the PROM change score, and the correlation between change scores and transition ratings. The calculation is defined as follows [26]:

$${\rm{MI}}{{\rm{C}}_{{\rm{Adjusted}}}} = {\rm{MI}}{{\rm{C}}_{\rm{Predictive}}} - \left( {{{0.8} \over {{\mathop{\rm Re}\nolimits} {{\rm{l}}_{{\rm{TR}}}}}} - 0.5} \right) * {\rm{S}}{{\rm{D}}_{{\rm{change}}}} * {\rm{Cor}} * \log \,{\rm{odds(imp)}} $$

Where is the improved adjusted MIC, $\:{\text{MIC}}_{\text{Predictive}}$ is the predictive MIC, $\:{\text{Rel}}_{\text{TR}}$ is the reliability of the transition ratings (here, our “Success” anchor), $\:{\text{SD}}_{\text{change}}$ is the standard deviation of the EQ-5D-3L change score, $\:\text{Cor}$ is the correlation between the EQ-5D-3L change score and the transition ratings, and $\:\text{log odds(imp)}$ represents the logit transformation of the proportion of improved patients. To estimate the reliability of transition ratings required for calculating the adjusted MIC, we employed a longitudinal confirmatory factor analysis (CFA) model with two time factors [41]. The model included the pre-operative measurement and the post-operative measurement as the two latent factors. As the CFA requires at least three items per factor, we employ the EQ-VAS as auxiliary variable (thanks to a correlation of 0.36 with the EQ-5D-3L). The reliability of the transition ratings was assessed by examining the R-square value of the transition rating item within the CFA model.    

As the aim of the MIC identification is to understand which EQ-5D-3L improvement corresponds to a meaningful improvement, the selected anchor was one question collected during the recording of PROMs for hip and knee replacements, called “Success” in the NHS PROMs dataset (“Overall, how are your problems now, compared to before your operation?”). Appendix, Table A3 shows that the majority of patients answer “Much better” to the question of the variable “Success”, followed by “A little better”. Since the Pearson correlation between the EQ-5D-3L change and the variable “Success” is -0.28 for hip replacements and − 0.32 for knee replacements, we conclude that this variable is suitable to be used as an anchor [42] once transformed into a binary variable (see Appendix, Table A2). Figures A2 and A3 in the Appendix additionally confirm the validity of the use of this variable as an anchor by showing the boxplots of the relationship between the EQ-5D-3L change and the variable “Success”. As we are looking at elective hip and knee replacement, we want to identify the smallest change in measurement that signifies an important improvement for the patient [43]. For this reason, we consider meaningfully improved those patients that reported their problems to be “Much better” or “A little better” with respect to before the surgery. The anchor “Success” was also used to estimate the MICs for sub-groups created according to depression status and sex.

To derive standard errors as a measure of precision for our MIC estimates, we applied the bootstrapping method with 1,000 resampled datasets, following the approach proposed by Terluin et al. [26]. The standard errors are calculated as the standard deviation of the bootstrapped samples. They are then multiplied by 1.96 and added and subtracted to the point estimates to obtain 95% confidence intervals.

Finally, we compared the size and precision of the unstratified and stratified MICs, as well as the share of patients achieving those thresholds.

All statistical analyses were performed using the statistical software R (version 4.3.2).

## Results

### Descriptive statistics

Table 1 displays the descriptive statistics for the MIC model. The majority of patients undergoing hip and knee replacement fell into the 60 to 79 age band (72.8% and 77.9%) and were female (60.7% and 56.9%). Most patients lived with their family (74.8% and 77.3%) and had symptoms for one to five years before the operation (69.7% and 52.3%). The most common comorbidity was arthritis (72.5% and 77.8%), followed by high blood pressure (38.1% and 44.7%). The mean pre-operative EQ-5D-3L index score for hip replacement patients was 0.350, lower than for knee replacement patients (0.417), but their mean post-operative EQ-5D-3L index score was 0.801, higher compared to knee replacement patients (0.746). Accordingly, hip replacement patients had a higher mean score improvement (0.451) compared to knee replacement patients (0.329). The same trend is true for the EQ-VAS, with mean pre-operative EQ-VAS for hip (knee) replacement patients being 64.403 (67.960) and post-operative EQ-VAS 77.672 (74.968).

The mean pre-operative OHS was 17.8, and the OKS was 19.1. As for the post-operative questionnaires, 95.8% (90.3%) of hip (knee) replacement patients answered the question “Overall, how are your problems now, compared to before your operation?” (variable “Success”) with “Much better” or “A little better”, 78.3% (63.5%) were satisfied with the results of the operation (variable “Satisfaction”), and 6% (7.8%) were readmitted (variable “Readmitted”).

**Table 1:** Descriptive statistics for the hip and knee replacement sample

	Hip			Knee		
	Observations	Mean/%	SD	Observations	Mean/%	SD
**Demographics**						
Age	232,824			261,115		
20 to 29		0.0%	-		0.0%	-
30 to 39		0.1%	-		0.0%	-
40 to 49		1.7%	-		0.2%	-
50 to 59		12.5%	-		10.2%	-
60 to 69		33.7%	-		36.1%	-
70 to 79		39.1%	-		41.8%	-
80 to 89		12.8%	-		11.7%	-
90 to 120		0.0%	-		0.0%	-
Gender	232,824			261,115		
Female		60.7	-		56.9%	-
Pre-operative living arrangements	248,941			275,433		
Living with partner/spouse/family/friends		74.8	-		77.3%	-
Living alone		24.7	-		22.2%	-
Living in a nursing home, hospital or other long term care home		0.1%	-		0.1%	-
Other		0.4%	-		0.3%	-
**Pre-operative patient health history**						
Pre-operative Assisted	250,411			277,443		
Yes		16.0	-		15.9%	-
Pre-operative Symptom Period	250,162			277,265		
Less than 1 year		12.4	-		4.6%	-
1 to 5 years		69.7	-		52.3%	-
6 to 10 years		11.7	-		22.1%	-
More than 10 years		6.2	-		21.0%	-
Pre-operative previous surgery	250,514			277,728		
Yes		3.6	-		3.9%	-
Pre-operative disability	238,548			268,054		
Yes		53.5	-		51.3%	-
**Comorbidities**	252,331			279,668		
Arthritis		72.5	-		77.8%	-
Cancer		5.4	-		5.3%	-
Circulation		4.6	-		5.8%	-
Depression		8.0	-		8.9%	-
Diabetes		9.2	-		12.6%	-
Heart disease		8.6	-		9.4%	-
High blood pressure		38.1	-		44.7%	-
Kidney disease		1.9	-		2.0%	-
Liver disease		0.6%	-		0.6%	-
Lung disease		8.2	-		9.1%	-
Nervous system		0.8	-		1.0%	-
Stroke		1.3	-		1.6%	-
**EQ-5D index score**						
Pre-op EQ-5D index score	252,331	0.35	0.32	279,668	0.417	0.309
[Median; Inter-quartile range]		[0.587; 0.101-0.691]			[0.516; 0.055-0.656]	
Post-op EQ-5D index score	252,331	0.801	0.24	279,668	0.746	0.249
[Median; Inter-quartile range]		[0.760; 0.689-1.000]			[0.848; 0.691-1.000]	
Post-op EQ-5D index score change	252,331	0.451	0.339	279,668	0.329	0.327
[Median; Inter-quartile range]		[0.309; 0.071-0.601]			[0.413; 0.204-0.736]	
**EQ-VAS**						
Pre-op EQ-VAS	230,608	64.403	21.978	254,508	67.960	20.142
Post-op EQ-VAS	230,608	77.672	17.593	254,508	74.968	17.987
Post-op EQ-VAS change	230,608	13.27	23.452	254,508	7.008	21.150
**Oxford Hip (Knee) Score**						
Hip (Knee) replacement pre-op score	249,827	17.829	8.06	276,654	19.124	7.663
Hip (Knee) replacement post-op score	250,420	39.987	8.493	277,661	36.001	9.367
Hip (Knee) replacement post-op score change	247,955	22.141	9.866	274,695	16.865	9.748
**Post-operative questionnaire**						
Post-op “Success”	252,331			279,668		
Yes		95.8	-		90.3%	-
Post-op “Satisfied”	251,449			278,391		
Yes		78.3%	-		63.5%	-
Post-op "Readmitted"	251,551			278,636		
Yes		6.0%	-		7.8%	-

SD = standard deviation

The pre-operative and post-operative EQ-5D-3L index score distributions remained rather constant over the years for both hip and knee replacement patients and do not present any trend (see Figures A4–A7 in the Appendix). Likewise, the sample size for both patient samples does not present unwarranted fluctuations. This shows that differences over the years do not explain variance in the scores, allowing to safely aggregate the observations over the years into a unitary dataset for the analyses.

### MICs

Subgroups of patients were created according to their pre-operative EQ-VAS scores. Table 2 reports the results of the MIC analysis using the anchor “Success”. The reliability of the transition ratings for the MIC model with the anchor “Success” was calculated through the CFA as 0.574 (0.590) for hip (knee) replacement patients. The estimated MIC value for hip (knee) replacement patients for the unstratified sample was 0.071 (0.046) for hip (knee) replacement patients, and the MICs varied among the different pre-operative EQ-VAS score subgroups. The MIC values ranged from 0.184 (0.147) for the patients in the subgroups with the lowest pre-operative EQ-VAS score (0 to 34 for hip replacement patients and 0 to 36 for knee replacement patients) to 0.014 for knee replacement patients with the highest pre-operative EQ-VAS score (87 to 100 for both hip and knee replacement patients) and 0.021 for hip replacement patients with pre-operative EQ-VAS score from 56 to 72. Generally, patients starting with worse pre-operative scores need larger improvements to reach their MIC. However, the middle pre-operative EQ-VAS subgroup is an exception, showing the lowest MIC.

The MICs calculated for knee replacement patients were consistently lower than those calculated for hip replacement patients for both unstratified and stratified samples.

**Table 2:** Unstratified and stratified MICs for hip and knee replacement according to the pre-operative EQ-VAS score

Hip replacement				Knee replacement			
	N	MIC	SE		N	MIC	SE
**Unstratified**	230,608	0.071	0.004	**Unstratified**	254,508	0.046	0.002
**Pre-operative EQ-VAS**				**Pre-operative EQ-VAS**			
0 to 34	25,964	0.184	0.010	0 to 36	21,270	0.147	0.007
35 to 55	50,620	0.102	0.008	37 to 56	47,087	0.075	0.005
56 to 72	57,831	0.021	0.007	57 to 72	65,708	0.026	0.004
73 to 86	59,230	0.044	0.007	73 to 86	72,988	0.017	0.004
87 to 100	36,963	0.053	0.010	87 to 100	47,455	0.014	0.006

N= number of observations; MIC = minimal important change; SE = standard error

**Fig. 1:**
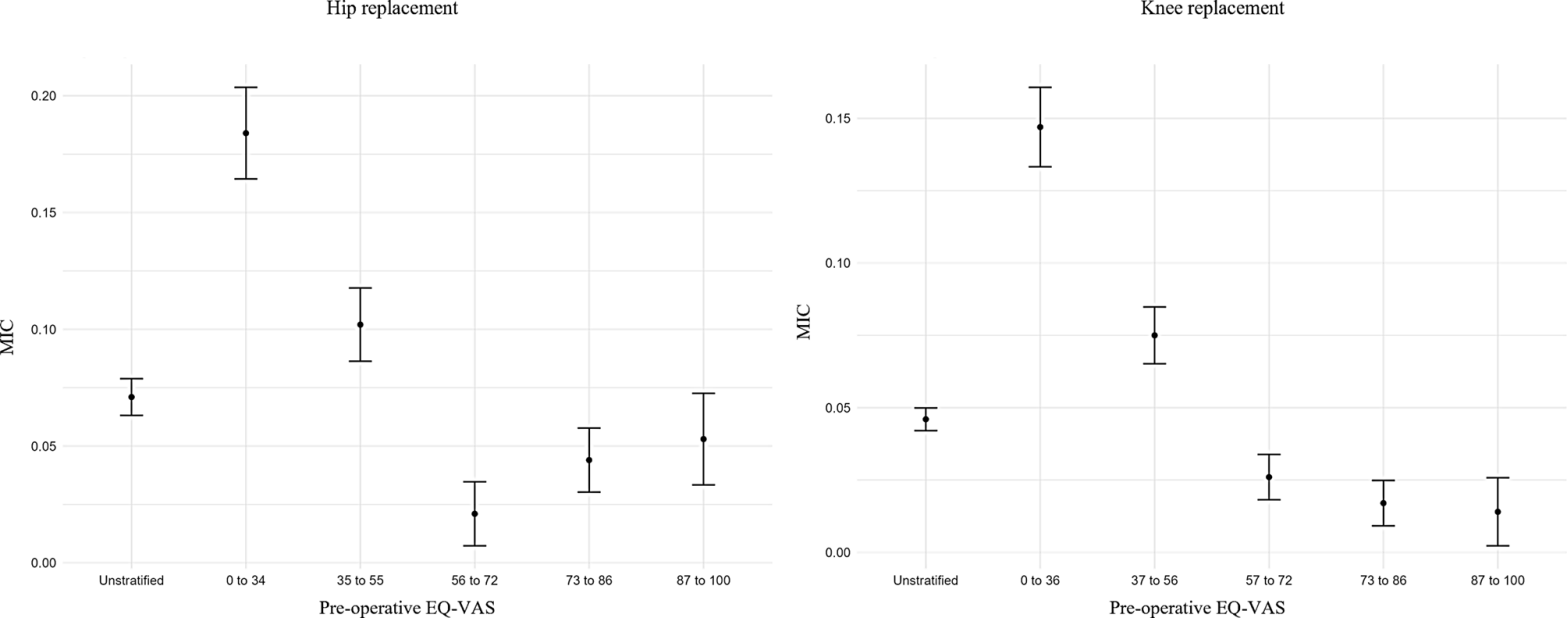
Confidence intervals for unstratified and stratified MICs for hip and knee replacement according to pre-operative EQ-VAS score

Figure 1 shows a visualization of the unstratified and stratified MIC estimates for hip and knee replacement patients, for which the same trends emerge. We notice how the confidence intervals are narrowest for the unstratified samples, which naturally present a larger sample size. We also observe how the MICs are starkly different between the first and second subgroup, and between the second subgroup and the three subgroups with highest pre-operative EQ-VAS. The MICs calculated for the subgroups with EQ-VAS from 56 onwards are instead similar.

Table 3 shows that hip replacement patients in the subgroup with pre-operative EQ-VAS from 56 to 72 that did not improve post-operatively have a much lower mean EQ-5D-3L change than the patients in the subgroups with higher pre-operative EQ-VAS. The mean EQ-5D-3L change in the improved groups is instead fairly similar between the subgroups with pre-operative EQ-VAS higher than 56. For knee replacement, instead, the mean EQ-5D-3L change for both the improved and the not improved patients is consistently higher in the subgroups with lower pre-operative EQ-VAS.

**Table 3:** EQ-5D-3L descriptives by EQ-VAS subgroups and by improvement status

Hip replacement						Knee replacement					
EQ-VAS subgroups	Impr.	N (%)	Mean pre-op EQ-5D	Mean post-op EQ-5D	Mean EQ-5D Change	EQ-VAS subgroups	Impr.	N (%)	Mean pre-op EQ-5D	Mean post-op EQ-5D	Mean EQ-5D Change
0 to 34	No	1304 (5%)	0.0472	0.254	0.207	0 to 36	No	2915 (14%)	0.101	0.258	0.157
	Yes	24,660 (95%)	0.0806	0.719	0.638		Yes	18,355 (86%)	0.156	0.652	0.495
35 to 55	No	2499 (5%)	0.264	0.390	0.127	37 to 56	No	5643 (12%)	0.292	0.384	0.092
	Yes	48,121 (95%)	0.278	0.776	0.498		Yes	41,444 (88%)	0.326	0.72	0.395
56 to 72	No	2484 (4%)	0.405	0.449	0.044	57 to 72	No	6573 (10%)	0.415	0.461	0.046
	Yes	55,347 (96%)	0.395	0.821	0.426		Yes	59,135 (90%)	0.437	0.774	0.338
73 to 86	No	2120 (4%)	0.431	0.493	0.062	73 to 86	No	5875 (8%)	0.477	0.516	0.039
	Yes	57,110 (96%)	0.440	0.858	0.418		Yes	67,113 (92%)	0.495	0.819	0.324
87 to 100	No	1077 (3%)	0.435	0.501	0.066	87 to 100	No	3336 (7%)	0.488	0.526	0.038
	Yes	35,886 (97%)	0.458	0.886	0.428		Yes	44,119 (93%)	0.517	0.850	0.332

*Note* “Impr.” Indicates whether the patients improved or not: “Yes” indicates patients who answered “a little better” or “much better” to the question from the variable “Success”; “No” indicates patients that replied otherwise; N = number of observations (percentages over the subgroup sample in brackets); EQ-5D = EQ-5D-3L

Table 4 shows that without adjusting for pre-operative EQ-VAS score the probability that a hip or knee replacement is meaningfully improved is higher for the patients group starting with worse EQ-VAS scores. It is possible to notice how, after the calculation of MICs stratified by pre-operative EQ-VAS score, a higher percentage of patients in the subgroups with higher pre-operative EQ-VAS achieve their stratified MIC. By looking at Table 3, one can see how this reflects the actual percentages of improved patients according to the post-operative question “Success”.

**Table 4:** MICs for hip and knee replacement patients – impact on the patient sample

Pre-operative EQ-VAS subgroups	EQ-5D-3L mean change	Unstratified MIC	% achieving unstratified MIC	Stratified MIC	% achieving stratified MIC
**Hip replacement**					
0 to 34	0.617	0.071	90%	0.184	85%
35 to 55	0.48	0.071	86%	0.102	85%
56 to 72	0.41	0.071	84%	0.021	89%
73 to 86	0.405	0.071	86%	0.044	89%
87 to 100	0.417	0.071	88%	0.053	90%
**Knee replacement**					
0 to 36	0.449	0.046	80%	0.147	72%
37 to 56	0.358	0.046	74%	0.075	73%
57 to 72	0.309	0.046	73%	0.026	81%
73 to 86	0.301	0.046	74%	0.017	80%
87 to 100	0.312	0.046	76%	0.014	81%

MIC = minimal important change

### Additional MIC stratifications

Table 5 shows additional stratifications of the MICs according to different patients’ characteristics.

We first explore the stratification of patients according to the variable “Depression” (i.e., having been told by a doctor to have depression), and according to “Depression” and “Pre-operative EQ-VAS” clustered together. The sub-group of patients with depression shows higher MICs for hip replacements (0.078) and knee replacements (0.069) when compared to those without depression (0.070 and 0.043 respectively) and compared to the unstratified MICs (0.071 and 0.046 respectively). However, the precision of the MIC estimates, as reflected in the standard errors (SE), is lower for the depressed subgroups in both hip and knee replacements, likely due to the smaller sample sizes. In contrast, the non-depression subgroup achieves higher precision, with smaller SEs for both hip and knee replacements that equal the SEs for the unstratified MICs, despite the smaller sample sizes.

When further stratifying by pre-operative EQ-VAS score, a reverse trend emerges. Throughout all pre-operative EQ-VAS subgroups, except for the knee replacement subgroup starting with highest pre-operative EQ-VAS, the MIC estimates for patients with depression are lower than the estimates for patients without depression. We also see that the proportion of patients with depression is higher in the lower pre-operative EQ-VAS score sub-groups compared to non-depressed patients, indicating a potential association between lower baseline health and depression.

We also examined MIC calculations stratified by sex. In both hip and knee replacements, males tend to have lower MICs than females, particularly in the knee replacement sample where the unstratified MIC for males is 0.027 compared to 0.064 for females. These differences persist across pre-operative EQ-VAS score sub-groups, with minor variations. For example, male hip replacement patients in the 0 to 34 score range show an MIC of 0.168, slightly lower than the 0.205 MIC for females in the same group. This suggests that male patients may perceive a meaningful improvement in their health status with smaller changes than females across most health states.

Overall, due to the larger sample size, the precision of the unstratified estimates is the largest across all subgroups.

**Table 5:** Unstratified and stratified MICs for hip and knee replacement according to “Pre-operative EQ-VAS score”, “Depression”, and “Gender”

Subgroups	Depression			No depression			Female			Male		
	N	MIC	SE	N	MIC	SE	N	MIC	SE	N	MIC	SE
**Hip replacement**												
**Unstratified**	18,475	0.078	0.011	212,133	0.07	0.004	127,529	0.083	0.005	85,008	0.056	0.006
**Pre-operative EQ-VAS**												
0 to 34	4,223	0.159	0.020	21,741	0.197	0.012	15,818	0.205	0.014	7,730	0.168	0.019
35 to 55	5,593	0.099	0.020	45,027	0.103	0.008	30,366	0.11	0.010	16,138	0.083	0.013
56 to 72	4,435	0.012	0.023	53,396	0.025	0.008	31,388	0.028	0.010	21,895	0.011	0.011
73 to 86	2,951	0.033	0.029	56,279	0.045	0.008	29,949	0.048	0.011	24,799	0.038	0.011
87 to 100	1,273	0.033	0.051	35,690	0.054	0.010	20,008	0.058	0.014	14,446	0.053	0.016
**Knee replacement**												
**Unstratified**	22,794	0.069	0.006	231,714	0.043	0.002	133,529	0.064	0.003	103,945	0.027	0.004
**Pre-operative EQ-VAS**												
0 to 36	4,414	0.157	0.011	16,856	0.147	0.008	12,792	0.161	0.010	6,659	0.143	0.012
37 to 56	6,276	0.072	0.012	40,811	0.077	0.005	28,316	0.088	0.007	15,461	0.059	0.008
57 to 72	6,128	0.026	0.013	59,580	0.026	0.005	34,859	0.041	0.006	26,507	0.009	0.007
73 to 86	4,173	0.004	0.017	68,815	0.018	0.005	34,382	0.033	0.007	33,906	0.006	0.006
87 to 100	1,803	0.056	0.026	45,652	0.012	0.006	23,180	0.032	0.009	21,412	0.003	0.008

N= number of observations; MIC = minimal important change; SE = standard error

## Discussion

With this study, we aimed at filling the research gap existing in the literature on MICs by estimating thresholds for the EQ-5D-3L index score reflecting meaningful improvements in the HRQoL of patients following hip or knee replacement, with some crucial innovations with respect to previous studies. Firstly, we leveraged the large sample size from the nationally representative NHS PROMs dataset. Secondly, the availability of external variables allowed us to calculate accurate MICs by selecting a reliable external anchor. In addition, the patient stratification was conducted through a machine-learning clustering algorithm considering the density distribution of the pre-operative EQ-VAS, allowing to identify meaningful patient clusters. Finally, we implemented a correction for the proportion of improved patients and the reliability of transition ratings, which have been shown to bias MIC estimates whenever the proportion of improved patients is higher than 50% [25, 26].

We show that by stratifying the sample based on patients’ pre-operative EQ-VAS score allows to uncover the differences in MICs depending on different patients’ characteristics. We find that patients starting with worse pre-operative scores need a larger improvement in the post-operative EQ-5D-3L index score to achieve a meaningful improvement. Literature has shown that low pre-operative PROM scores are associated with high pre-operative expectations [21, 44]. While this fact can potentially play a role in explaining our findings, the relationship between pre-operative expectations and post-operative outcomes and MICs is still unclear [45–47]. We also notice that, while differences in MICs are marked between the lowest two pre-operative EQ-VAS score subgroups and between these and the highest three pre-operative EQ-VAS score subgroups, MICs for the latter are fairly similar. This suggests that the three hip (knee) replacement subgroups with EQ-VAS from 56 (57) onwards could be considered together when calculating the MICs.

In addition, we show that depressed patients exhibit higher MICs for both hip and knee replacements compared to non-depressed patients, but present smaller MICs across all pre-operative EQ-VAS subgroups. This can be explained by the fact that a higher proportion of depressed patients fall into the lower pre-operative EQ-VAS subgroups. This aligns with expectations, given that depression and anxiety are captured by the EQ-5D-3L, which includes mental health dimensions [48]. As such, depressed patients tend to report lower baseline health status. Our finding exemplifies how failing to stratify this sample by pre-operative health status would therefore lead to biased results.

Finally, we show that male patients exhibit lower MICs than females. These differences persist across pre-operative EQ-VAS groups, suggesting that males may perceive meaningful improvements with smaller health status changes than females across various baseline health levels. As notably female patients tend to start with worse pre-operative EQ-VAS scores [49, 50], this further confirms the necessity to adjust for patients’ characteristics.

We also show how with respect to the percentage of patients achieving their unstratified MIC, the percentage of patients achieving their stratified MIC is better in line with the actual share of improved patients according to our anchor variable.

Overall, our findings show that the use of unstratified thresholds to evaluate treatment outcomes or detect critical recovery paths are not advisable and would lead to an inaccurate assessment of hip and knee replacement successes. MIC values calculated based on patients’ EQ-VAS scores at admission, instead, provide thresholds that are better tailored to individual patients and more reflective of actual post-surgery improvements.

### Findings from other studies

While several studies attempted to estimate (unstratified) MICs based on PROMs for hip and knee replacement patients, and despite several studies emphasizing that the share of patients achieving an unstratified MIC depends on their admission score [51–53], literature on thresholds adjusted for patient characteristics is relatively limited [54]. To the best of our knowledge, no study has so far estimated MICs for hip and knee replacement patients clustered according to both depression status and pre-operative PROMs, and according to sex and pre-operative PROMs. Davis et al. [55] showed that unstratified thresholds to assess treatment outcome after orthopedic surgery led to an unfair assessment of successful outcomes, depending on pre-operative PROM scores. Paulsen et al. [24] and Kuklinski et al. [22] estimate MIC values based on subgroups clustered by pre-operative PROM scores. Through a distribution-based method, Paulsen et al. [24] showed that MCID values vary significantly among subgroups of patients with different pre-operative PROM scores (lowest tertile: 0.67; middle tertile: 0.34; highest tertile: 0.23). Through an anchor-based method, Kuklinski et al. [22] showed that thresholds for meaningful improvement in PROM scores need to be adjusted to patient characteristics and that MCID thresholds for hip replacement at 3-months (12-months) follow-up are 0.637 (0.889) for the lowest pre-operative EQ-5D-3–5 L group, and 0.001 (0.113) for the highest pre-operative score group. Gutacker et al. [56] also employ the NHS PROMs datasets to demonstrate the importance of patient stratification when classifying hip and knee replacements.

Thanks to the adjustment for the proportion of improved patients and the reliability of transition ratings, our results for unstratified and stratified MIC thresholds are notably lower than the MICs found in existing literature. This is explained by the fact that unadjusted MIC estimates are overestimated in datasets with more than 50% of patients improved on the anchor [25, 26]. As the NHS PROMs datasets have around 90% of patients improved according to the anchor “Success”, adjustment for the proportion of improved patients and reliability of the anchor is necessary.

### Limitations

Since PROMs information comes from survey data, our dataset may suffer from a problem of responder bias, as patients with extremely poor recoveries may not be able to answer the questionnaire, even if assisted. However, the English ‘best practice tariff’ pay-for-performance scheme creates an incentive for providers to meet the minimum standard for data collection [57, 58].

Another limitation comes from the presence of ceiling effects in the EQ-5D-3L [59]. Many observations had relatively high pre-operative scores and few hundred observations had to be discarded due to patients already having reached the perfect health score pre-operatively. However, the patient stratification that we implemented based on the pre-operative EQ-VAS score effectively separated patients starting with low and high pre-operative scores, allowing to provide better-tailored MIC estimates.

One limitation connected to the anchor-based methodology lies in the fact that for hip replacements, the correlation between the “Success” variable and the EQ-5D-3L change is marginally below the recommended 0.3 threshold, which might lead to decreased precision in our estimates [60]. However, the large sample size in our model ensures a high level of precision for the MIC estimates. Furthermore, the reliability of our anchor with respect to measuring the EQ-5D-3L change is relatively high compared with values for transition ratings found in the literature [61].

Anchor-based methods have also been criticized for the effect of recall bias, or better, present state bias, on long-term responsiveness and for their inability to include the measurement precision of the global instrument [16]. However, it has been shown that present state bias does not impact MICs estimated through the predictive modeling method [62].

## Conclusions

Our research shows that when evaluating PROM results by using MICs, they should be adjusted for patients’ characteristics, and especially for pre-operative PROM scores, as patients starting with worse pre-operative scores need larger improvements for surgeries to be considered successful. By doing so, a more accurate evaluation of surgery successes can be achieved.

We envision our findings to be used as inputs for the refinement of clinical decision support systems, thus benefitting several groups of stakeholders in the healthcare system. Physicians could take our findings into consideration when monitoring patients’ recovery paths to enable a reliable feedback cycle and treatment path adaption. When using PROMs for quality monitoring and policy design, health policy makers might consider our findings to assess the quality of hospital treatments independently from patient selection bias. Our findings can also guide health insurers in the development of pay-for-performance contracts based on surgery success evaluated through PROMs. Likewise, patients could benefit from exposure to these findings for better hospital choices based on expected surgery success.

Future research should aim at providing MIC thresholds that are adjusted for patients’ characteristics and as precise as possible. Furthermore, future research would benefit from the exploration of MICs for disease-specific instruments, such as the OHS and OKS, in addition to MICs for the generic instrument EQ-5D.

## Electronic supplementary material

Below is the link to the electronic supplementary material.

Supplementary Material 1

## Data Availability

The data used for the analyses in this manuscript are publicly available (at this website: https://digital.nhs.uk/data-and-information/publications/statistical/patient-reported-outcome-measures-proms), and were made available by NHS England. We further detail in the electronic online supplement the process with which the data was accessed and merged.

## References

[1] Deakin AH, Smith MA, Wallace DT et al (2019) Fulfilment of preoperative expectations and postoperative patient satisfaction after total knee replacement. A prospective analysis of 200 patients. Knee 26:1403–1412. 10.1016/J.KNEE.2019.07.018 31474421 10.1016/j.knee.2019.07.018

[2] hips-all-procedures-activity https://reports.njrcentre.org.uk/hips-all-procedures-activity/K01v2NJR?reportid=C6F582E2-140D-4D22-8C4E-2C354EDB1B41&defaults=DC__Reporting_Period__Date_Range=%222020%7CNJR2019%22,JYS__Filter__Calendar_Year__From__To=%22max-max%22,H__Filter__Joint=%22Knee%22. Accessed 29 Jul 2022

[3] knees-all-procedures-activity https://reports.njrcentre.org.uk/knees-all-procedures-activity. Accessed 29 Jul 2022

[4] OECD (2021) Health at a Glance 2021. 10.1787/AE3016B9-EN

[5] Ray G, Ekelund P, Nemes S et al (2019) Changes in health-related quality of life are associated with patient satisfaction following total hip replacement: an analysis of 69,083 patients in the Swedish hip. Taylor Francis 91:48–52. 10.1080/17453674.2019.1685284 10.1080/17453674.2019.1685284 PMC7008235 31680594

[6] Norman-Taylor FH, Palmer CR, Villar RN (1996) Quality-of-life improvement compared after hip and knee replacement. J Bone Joint Surg - Ser B 78:74–77. 10.1302/0301-620X.78B1.0780074 8898131

[7] Ney JP, Taylor LP (2019) Patients are the best judges. Neurol Clin Pract 9:7–8. 10.1212/CPJ.0000000000000572 30859001 10.1212/CPJ.0000000000000572 PMC6382380

[8] Padilla G v, Rhiner M, Bogen C (1992) Health quality of life and colorectal cancer. 10.1002/1097-0142 10.1002/1097-0142(19920901)70:3+<1450::aid-cncr2820701537>3.0.co;2-e 1511396

[9] Patient-reported outcomes in acute care | Health at a Glance (2023): OECD Indicators | OECD iLibrary. https://www.oecd-ilibrary.org/sites/03d264a2-en/index.html?itemId=/content/component/03d264a2-en. Accessed 22 Mar 2024

[10] Gwynne-Jones DP, Sullivan T, Wilson R, Abbott JH (2020) The Relationship between Preoperative Oxford hip and knee score and change in Health-Related Quality of Life after total hip and total knee arthroplasty: can it help inform rationing decisions? Arthroplast Today 6:585–589e1. 10.1016/J.ARTD.2020.04.009 32995405 10.1016/j.artd.2020.04.009 PMC7502579

[11] Hurst NP, Kind P, Ruta D et al (1997) Measuring health-related quality of life in rheumatoid arthritis: validity, responsiveness and reliability of EuroQol (EQ-5D). Rheumatology 36:551–559. 10.1093/RHEUMATOLOGY/36.5.551 10.1093/rheumatology/36.5.551 9189057

[12] Shao Z, Bi S (2022) Patient satisfaction after total hip arthroplasty: influencing factors. Front Surg 9. 10.3389/FSURG.2022.1043508 10.3389/fsurg.2022.1043508 PMC9922864 36793514

[13] Sabah SA, Knight R, Alvand A et al (2022) Early patient-reported outcomes from primary hip and knee arthroplasty have improved over the past seven years: an analysis of the NHS PROMs dataset. Bone Joint J 104–B:687–695. 10.1302/0301-620X.104B6.BJJ-2021-1577.R1 35638211 10.1302/0301-620X.104B6.BJJ-2021-1577.R1

[14] Choi Y-J, Ra HJ (2016) Patient satisfaction after total knee arthroplasty. Knee Surg Relat Res 28:1–15. 10.5792/ksrr.2016.28.1.1 26955608 10.5792/ksrr.2016.28.1.1 PMC4779800

[15] Statistics » Patient Reported Outcome Measures (PROMs) https://www.england.nhs.uk/statistics/statistical-work-areas/proms/. Accessed 29 Jul 2022

[16] Wright A, Hannon J, Hegedus EJ, Kavchak AE (2012) Clinimetrics corner: a closer look at the minimal clinically important difference (MCID). J Man Manip Ther 20:160–166. 10.1179/2042618612Y.0000000001 23904756 10.1179/2042618612Y.0000000001 PMC3419574

[17] Page P (2014) Beyond statistical significance: clinical interpretation of rehabilitation research literature. Int J Sports Phys Ther 9:726 25328834 PMC4197528

[18] Batterham AM, Hopkins WG (2006) Making meaningful inferences about magnitudes. Int J Sports Physiol Perform 1:50–57. 10.1123/IJSPP.1.1.50 19114737

[19] Kristensen N, Nymann C, Konradsen H (2016) Implementing research results in clinical practice- the experiences of healthcare professionals. BMC Health Serv Res 16. 10.1186/S12913-016-1292-Y 10.1186/s12913-016-1292-y PMC4748469 26860594

[20] Terwee CB, Peipert JD, Chapman R et al (2021) Minimal important change (MIC): a conceptual clarification and systematic review of MIC estimates of PROMIS measures. Qual Life Res 30:2729. 10.1007/S11136-021-02925-Y 34247326 10.1007/s11136-021-02925-y PMC8481206

[21] Farrow L, Redmore J, Talukdar P et al (2022) Prioritisation of patients awaiting hip and knee arthroplasty: lower pre-operative EQ-5D is associated with greater improvement in quality of life and joint function. Musculoskelet Care. 10.1002/MSC.1645 10.1002/msc.1645 PMC10084259 35560766

[22] Kuklinski D, Marques CJ, Bohlen K et al (2022) Thresholds for meaningful improvement in WOMAC scores need to be adjusted to patient characteristics after hip and knee replacement. J Orthop 29:50–59. 10.1016/J.JOR.2022.01.002 35125779 10.1016/j.jor.2022.01.002 PMC8803617

[23] Giesinger JM, Hamilton DF, Jost B et al (2015) WOMAC, EQ-5D and Knee Society Score Thresholds for Treatment Success after Total Knee Arthroplasty. J Arthroplasty 30:2154–2158. 10.1016/J.ARTH.2015.06.012 26160647 10.1016/j.arth.2015.06.012

[24] Paulsen A, Roos EM, Pedersen AB, Overgaard S (2014) Minimal clinically important improvement (MCII) and patient-acceptable symptom state (PASS) in total hip arthroplasty (THA) patients 1 year postoperatively. Acta Orthop 85:39–48. 10.3109/17453674.2013.867782/SUPPL_FILE/IORT_A_867782_SM0001.PDF 24286564 10.3109/17453674.2013.867782 PMC3940990

[25] Terluin B, Eekhout I, Terwee CB (2017) The anchor-based minimal important change, based on receiver operating characteristic analysis or predictive modeling, may need to be adjusted for the proportion of improved patients. J Clin Epidemiol 83:90–100. 10.1016/J.JCLINEPI.2016.12.015 28093262 10.1016/j.jclinepi.2016.12.015

[26] Terluin B, Eekhout I, Terwee CB (2022) Improved adjusted minimal important change took reliability of transition ratings into account. J Clin Epidemiol 148:48–53. 10.1016/J.JCLINEPI.2022.04.018 35436522 10.1016/j.jclinepi.2022.04.018

[27] Velentgas P, Dreyer NA, Nourjah P et al (2013) Developing a protocol for Observational comparative effectiveness research: a user’s guide. Developing a protocol for Observational comparative effectiveness research: a user’s guide, pp 177–184 23469377

[28] Briggs AM, Woolf AD, Dreinhöfer K et al (2018) Reducing the global burden of musculoskeletal conditions. Bull World Health Organ 96:366. 10.2471/BLT.17.204891 29875522 10.2471/BLT.17.204891 PMC5985424

[29] Hertler C, Seiler A, Gramatzki D et al (2020) Sex-specific and gender-specific aspects in patient-reported outcomes. ESMO Open 5. 10.1136/ESMOOPEN-2020-000837 10.1136/esmoopen-2020-000837 PMC7662538 33184099

[30] Finalised Patient Reported Outcome Measures (PROMs) in England for Hip and Knee Replacement Procedures (April 2019 to March 2020) - NHS Digital. https://digital.nhs.uk/data-and-information/publications/statistical/patient-reported-outcome-measures-proms/finalised-hip-and-knee-replacement-april-2019---march-2020. Accessed 3 Nov 2023

[31] Von Elm E, Altman DG, Egger M et al (2009) The strengthening the reporting of observational studies in epidemiology (STROBE) statement: guidelines for reporting observational studies. UroToday Int J 2. 10.4038/jccpsl.v13i2.2965 10.1136/bmj.39335.541782.AD PMC2034723 17947786

[32] National Health Service (NHS) Digital (2022) Background information about PROMs. https://digital.nhs.uk/data-and-information/data-tools-and-services/data-services/patient-reported-outcome-measures-proms/background-information-about-proms. Accessed 27 Jul 2022

[33] Dolan P, Gudex C, Kind P, Williams A (1995) A social tariff for EuroQol. results from a UK general population survey

[34] Dawson J, Fitzpatrick R, Carr A, Murray D (1996) Questionnaire on the perceptions of patients about total hip replacement. J Bone Joint Surg - Ser B 78:185–190. 10.1302/0301-620X.78B2.0780185/LETTERTOEDITOR 8666621

[35] Dawson J, Fitzpatrick R, Murray D, Carr A (1998) Questionnaire on the perceptions of patients about total knee replacement. J Bone Joint Surg Br 80:63–69. 10.1302/0301-620X.80B1.7859 9460955 10.1302/0301-620x.80b1.7859

[36] Terluin B, Roos EM, Terwee CB et al (2021) Assessing baseline dependency of anchor-based minimal important change (MIC): don’t stratify on the baseline score! Qual Life Res 30:2773–2782. 10.1007/S11136-021-02886-2 34041680 10.1007/s11136-021-02886-2 PMC8481187

[37] Patient Reported Outcome Measures (PROMs) - NHS England Digital. https://digital.nhs.uk/data-and-information/data-tools-and-services/data-services/patient-reported-outcome-measures-proms#guidance. Accessed 4 Oct 2024

[38] Guyatt G, Walter S, Norman G (1987) Measuring change over time: assessing the usefulness of evaluative instruments. J Chronic Dis 40:171–178. 10.1016/0021-9681(87)90069-5 3818871 10.1016/0021-9681(87)90069-5

[39] Jaeschke R, Singer J, Guyatt GH (1989) Measurement of health status: ascertaining the minimal clinically important difference. Control Clin Trials 10:407–415. 10.1016/0197-2456(89)90005-6 2691207 10.1016/0197-2456(89)90005-6

[40] Crosby RD, Kolotkin RL, Williams GR (2003) Defining clinically meaningful change in health-related quality of life. J Clin Epidemiol 56:395–407. 10.1016/S0895-4356(03)00044-1 12812812 10.1016/s0895-4356(03)00044-1

[41] Griffiths P, Terluin B, Trigg A et al (2022) A confirmatory factor analysis approach was found to accurately estimate the reliability of transition ratings. J Clin Epidemiol 141:36–45. 10.1016/J.JCLINEPI.2021.08.029 34464687 10.1016/j.jclinepi.2021.08.029

[42] (2011) A point of minimal important difference (MID): a critique of terminology and methods. 10.1586/ERP.11.9 10.1586/erp.11.9 21476819

[43] Kvien TK, Heiberg T, Hagen KB (2007) Minimal clinically important improvement/difference (MCII/MCID) and patient acceptable symptom state (PASS): what do these concepts mean? Ann Rheum Dis 66:iii40–iii41. 10.1136/ARD.2007.079798 17934093 10.1136/ard.2007.079798 PMC2095292

[44] Scott CEH, Bugler KE, Clement ND et al (2012) Patient expectations of arthroplasty of the hip and knee. J Bone Joint Surg - Ser B 94 B:974–981. 10.1302/0301-620X.94B7.28219/ASSET/IMAGES/LARGE/28219-GALLEYFIG1B.JPEG. 10.1302/0301-620X.94B7.28219 22733956

[45] Waljee J, McGlinn EP, Sears ED, Chung KC (2014) Patient expectations and patient-reported outcomes in surgery: a systematic review. Surgery 155:799–808. 10.1016/J.SURG.2013.12.015 24787107 10.1016/j.surg.2013.12.015 PMC4170731

[46] Jacob KC, Patel MR, Collins AP et al (2022) Meeting patient expectations and achieving a minimal clinically important difference for back disability, back Pain, and Leg Pain May provide predictive utility for achieving patient satisfaction among lumbar decompression patients. World Neurosurg 162:e328–e335. 10.1016/J.WNEU.2022.03.002 35259504 10.1016/j.wneu.2022.03.002

[47] Chahla J, Beck EC, Nwachukwu BU et al (2019) Is there an Association between Preoperative expectations and patient-reported Outcome after Hip Arthroscopy for Femoroacetabular Impingement Syndrome? Arthroscopy: J Arthroscopic Relat Surg 35:3250–3258e1. 10.1016/J.ARTHRO.2019.06.018 10.1016/j.arthro.2019.06.018 31785753

[48] Van Reenen M, Janssen B, Stolk E et al (2019) EQ-5D-5L User Guide

[49] Lim JBT, Chi CH, Lo LE et al (2015) Gender difference in outcome after total knee replacement. J Orthop Surg 23:194–197. 10.1177/230949901502300216 10.1177/230949901502300216 26321549

[50] Katz J, Wright E,… EG-…: OJ of, 1994 undefined (1994) Differences between men and women undergoing major orthopedic surgery for degenerative arthritis. Wiley Online LibraryJN Katz, EA Wright, E Guadagnoli, MH Liang, EW Karlson, PD ClearyArthritis & Rheumatism: Official Journal of the American College, 1994•Wiley Online Library 37:687–694. 10.1002/art.1780370512 10.1002/art.1780370512 8185695

[51] Berliner JL, Brodke DJ, Chan V et al (2017) Can Preoperative patient-reported outcome measures be used to predict meaningful improvement in function after TKA? Clin Orthop Relat Res 475:149–157. 10.1007/s11999-016-4770-y 26956248 10.1007/s11999-016-4770-y PMC5174023

[52] Berliner JL, Brodke DJ, Chan V et al (2016) John Charnley Award: preoperative patient-reported Outcome measures Predict clinically meaningful improvement in function after THA. Clin Orthop Relat Res 474:321–329. 10.1007/s11999-015-4350-6 26201420 10.1007/s11999-015-4350-6 PMC4709271

[53] Most J, Hoelen TCA, Spekenbrink-Spooren A et al (2022) Defining clinically meaningful thresholds for patient-reported outcomes in knee arthroplasty. J Arthroplasty 37:837–844e3. 10.1016/J.ARTH.2022.01.092 35134515 10.1016/j.arth.2022.01.092

[54] Rouquette A, Blanchin M, Sébille V et al (2014) The minimal clinically important difference determined using item response theory models: an attempt to solve the issue of the association with baseline score. J Clin Epidemiol 67:433–440. 10.1016/j.jclinepi.2013.10.009 24447591 10.1016/j.jclinepi.2013.10.009

[55] Davis AM, Perruccio AV, Lohmander LS (2012) Minimally clinically important improvement: all non-responders are not really non-responders an illustration from total knee replacement. Osteoarthritis Cartilage 20:364–367. 10.1016/j.joca.2012.02.005 22343574 10.1016/j.joca.2012.02.005

[56] Gutacker N, Street A (2017) Use of large-scale HRQoL datasets to generate individualised predictions and inform patients about the likely benefit of surgery. Qual Life Res 26:2497–2505. 10.1007/S11136-017-1599-0 28567601 10.1007/s11136-017-1599-0 PMC5548850

[57] Gomes M, Gutacker N, Bojke C, Street A (2016) Addressing Missing Data in patient-reported outcome measures (PROMS): implications for the use of PROMS for comparing provider performance. Health Econ 25:515–528. 10.1002/HEC.3173 25740592 10.1002/hec.3173 PMC4973682

[58] Consultation on 2021/22 National Tariff Payment System

[59] Brazier J, Roberts J, Tsuchiya A, Busschbach J (2004) A comparison of the EQ-5D and SF-6D across seven patient groups. Health Econ 13:873–884. 10.1002/HEC.866 15362179 10.1002/hec.866

[60] Revicki D, Hays RD, Cella D, Sloan J Recommended methods for determining responsiveness and minimally important differences for patient-reported outcomes. 10.1016/j.jclinepi.2007.03.012 10.1016/j.jclinepi.2007.03.012 18177782

[61] Griffiths P, Terluin B, Trigg A,… WS-J of C (2022) A confirmatory factor analysis approach was found to accurately estimate the reliability of transition ratings. ElsevierP Griffiths, B Terluin, A Trigg, W Schuller, JB BjornerJournal of Clinical Epidemiology, 2022•Elsevier 10.1016/j.jclinepi.2021.08.029 34464687

[62] Terluin B, Fromy P, Trigg A et al (2024) Effect of present state bias on minimal important change estimates: a simulation study. Qual Life Res. 10.1007/S11136-024-03763-4 39174866 10.1007/s11136-024-03763-4 PMC11541299

