# Supplementary material for: How to interpret patient-reported outcomes? - Stratified adjusted minimal important changes for the EQ-5D-3L in hip and knee replacement patients
Source: J Patient Rep Outcomes. 2024 Nov 25;8:136. doi: 10.1186/s41687-024-00812-x (PMC11589056; doi:10.1186/s41687-024-00812-x)

**Appendix for the manuscript “How to interpret patient-reported outcomes? - Stratified adjusted minimal important changes for the EQ-5D-3L in hip and knee replacement patients” submitted to *Journal of Patient-Reported Outcomes***

**Table A1. Description of data access procedure**

| **Step** | **Procedure** |
| --- | --- |
| 1 | [Visit the main website: https://digital.nhs.uk/data-and-information/publications/statistical/patient-reported-outcome-measures-proms](https://digital.nhs.uk/data-and-information/publications/statistical/patient-reported-outcome-measures-proms) |
| 2 | [From the main website, select "Finalised Patient Reported Outcome Measures (PROMs) in England for Hip & Knee Replacements, April 20XX – March 20XX" links for each year (in our case from 2013 to 2020). For example, for 2019 to 2020 the link is: https://digital.nhs.uk/data-and-information/publications/statistical/patient-reported-outcome-measures-proms/finalised-hip-and-knee-replacement-april-2019---march-2020](https://digital.nhs.uk/data-and-information/publications/statistical/patient-reported-outcome-measures-proms/finalised-hip-and-knee-replacement-april-2019---march-2020) |
| 3 | Under “Resources”, download the "CSV Data Pack" files for each year |
| 4 | Within the downloaded folder, select "Record Level Hip Replacement XXXX" and "Record Level Knee Replacement XXXX" (2013 to 2015), or "Hip Replacement XXXX" and "Knee Replacement XXXX" (2015-2016), or "Hip Replacement Provider XXXX" and "Knee Replacement Provider XXXX" (2016-2020) |
| 5 | Merge the selected files in one dataset from 2013 to 2020. |

**Figure A1. Data cleaning process**


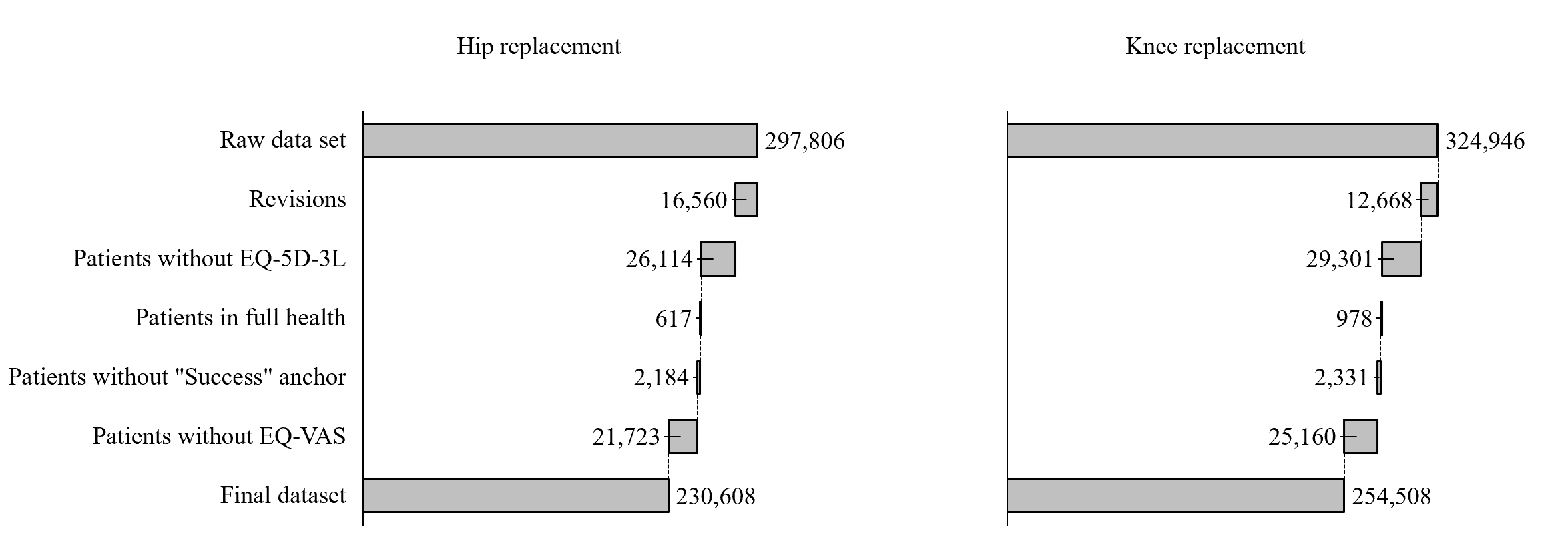


Note: “Patients without EQ-5D-3L (EQ-VAS)“ indicates observations missing either pre-operative or post-operative EQ-5D-3L (EQ-VAS) questionnaire. “Patients in full health” indicates observations for which the EQ-5D-3L index score = 1.

**Figure A2. Boxplot for the relationship between EQ-5D-3L Index Change and variable “Success” for hip replacement sample**

**
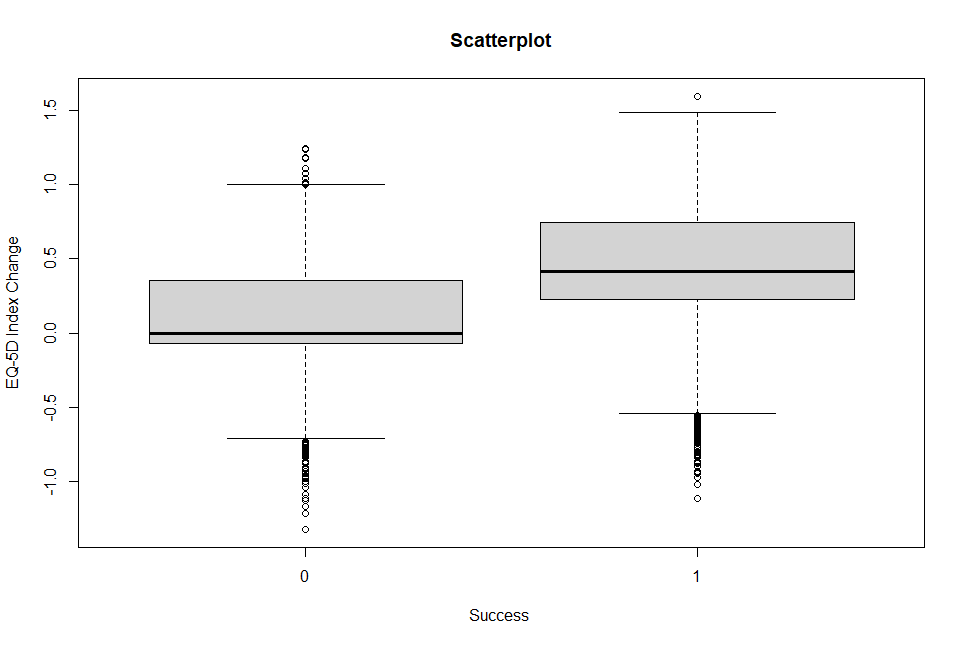
**

**Figure A3. Box plot for the relationship between EQ-5D-3L Index Change and variable “Success” for knee replacement sample**

**
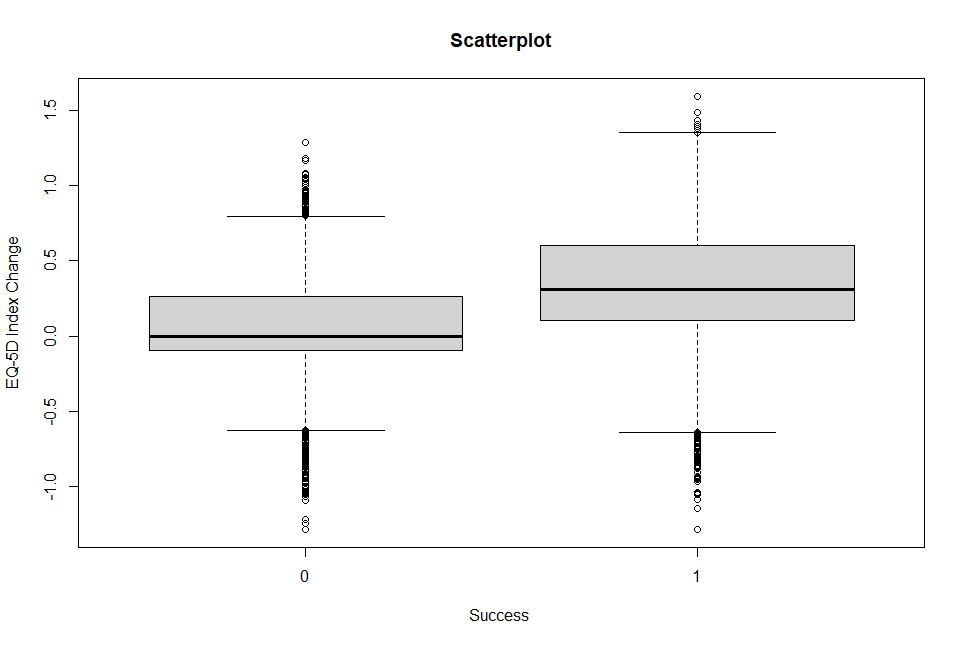
**

**Table A2. Variable transformation**

| **Variable name** | **NHS dataset variables** | **Recoded variables** |
| --- | --- | --- |
| **Gender** | 1= Male | 0 = Male |
|  | 2 = Female | 1 = Female |
| **Comorbidities** | 1= Yes | 1 = Yes |
|  | 9 = Missing | 0 = No |
| **Success** | 1 = Much better | Yes |
|  | 2 = A little better |  |
|  | 3 = About the same | No |
|  | 4 = A little worse |  |
|  | 5 = Much worse |  |
| **Satisfaction** | 1 = Excellent | Yes |
|  | 2 = Very Good |  |
|  | 3 = Good | No |
|  | 4 = Fair |  |
|  | 5 = Poor |  |

**Table A3. Percentage of answers to the variable “Success”**

| **Descriptive Statistics Hip (Knee) Replacement** | | | | | | |
| --- | --- | --- | --- | --- | --- | --- |
|  | Hip | | | Knee | | |
|  | **Observations** | **Mean/%** | **SD** | **Observations** | **Mean/%** | **SD** |
| **Variable "Success"** | 252331 |  |  | 279,668 |  |  |
| Much better |  | 87.8% | - |  | 74.9% | - |
| A little better |  | 8.0% | - |  | 15.4% | - |
| About the same |  | 2.1% | - |  | 4.3% | - |
| A little worse |  | 1.2% | - |  | 3.3% | - |
| Much worse |  | 0.9% | - |  | 2.1% | - |

**Figure A4. Evolution of pre-operative EQ-5D-3L index score – hip replacement sample**


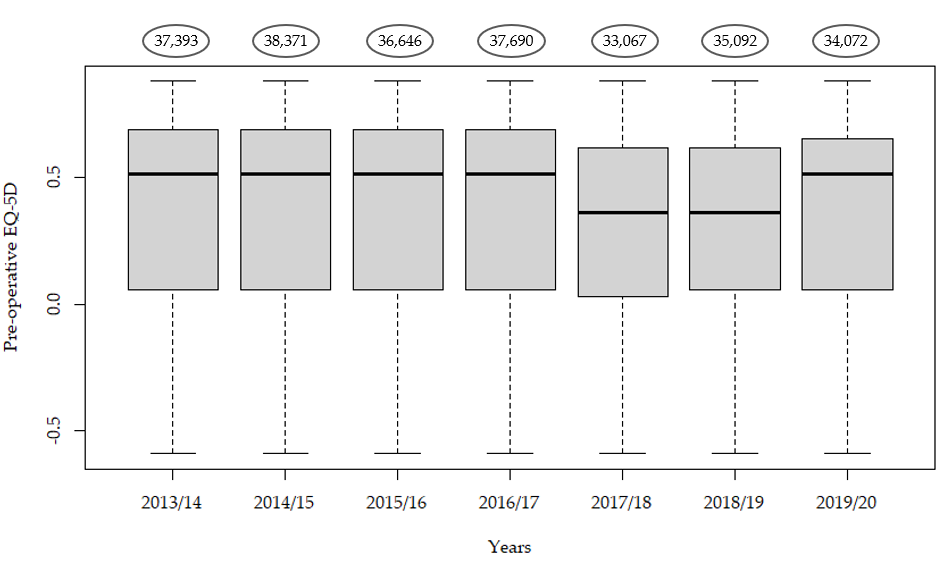


**Figure A5. Evolution of pre-operative EQ-5D-3L index score – knee replacement sample**


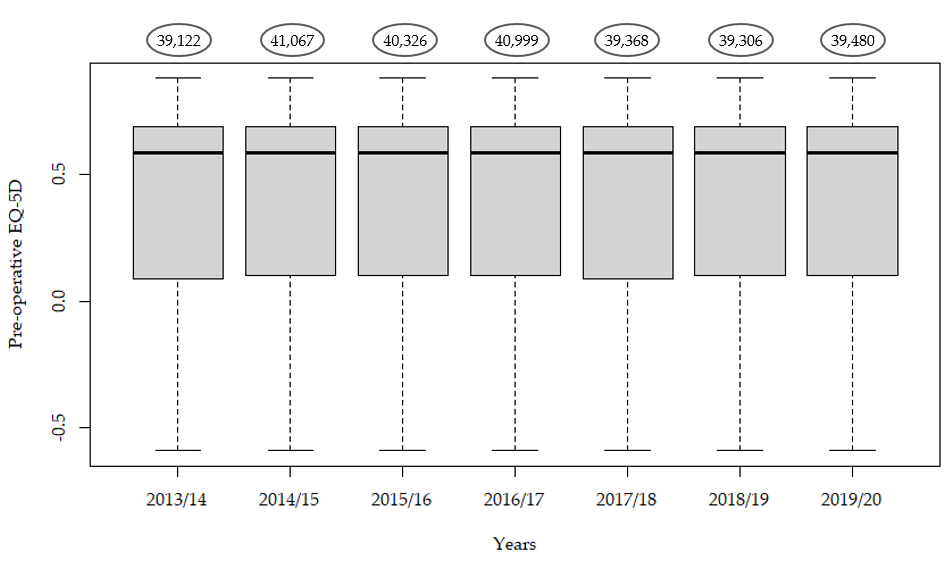


**Figure A6. Evolution of post-operative EQ-5D-3L index score – hip replacement sample**


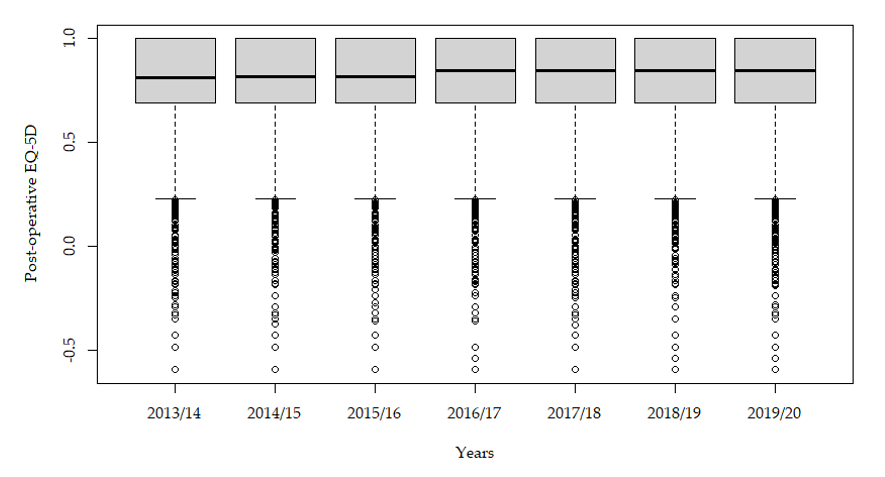


**Figure A7. Evolution of post-operative EQ-5D-3L index score – knee replacement sample**


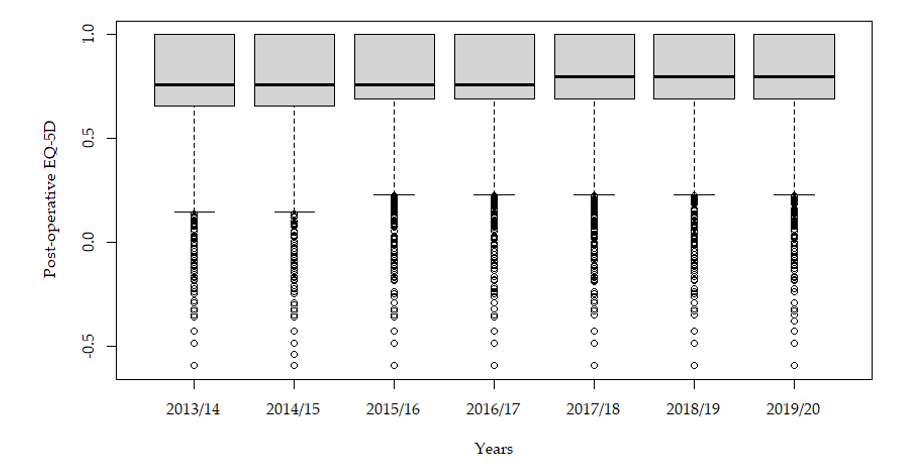

Supplement: Supplementary file 1 — Supplementary Material 1 [file 41687_2024_812_MOESM1_ESM.docx]
